# Psychological Correlates of Excessive Healthy and Orthorexic Eating: Emotion Regulation, Attachment, and Anxious-Depressive-Stress Symptomatology

**DOI:** 10.3389/fnut.2022.817047

**Published:** 2022-03-09

**Authors:** Jana Strahler, Hanna Wachten, Shanna Neuhofer, Peter Zimmermann

**Affiliations:** ^1^Department of Sport Psychology, Albert-Ludwigs-University Freiburg, Freiburg im Breisgau, Germany; ^2^Department of Health Psychology and Applied Diagnostics, University of Wuppertal, Wuppertal, Germany; ^3^Department of Psychotherapy and System Neuroscience, Justus Liebig University Giessen, Giessen, Germany; ^4^Department of Developmental Psychology, University of Wuppertal, Wuppertal, Germany

**Keywords:** healthful eating, orthorexia nervosa, emotion regulation, attachment, affective psychopathology, stress

## Abstract

Orthorexia nervosa, the pathological obsession with eating healthy, shares risks and significant comorbidity with other mental disorders. Based on a behavioral conceptualization of the overlap, emotion regulation, attachment style, and anxious-depressive-stress symptomatology are prominent but insufficiently researched endophenotypes for orthorexia nervosa. This study aimed at identifying ways in which difficulties in emotion regulation and attachment-related anxiety and avoidance become apparent in orthorexia nervosa and healthy orthorexia. Additionally, the moderating role of anxious, depressive, and stress symptoms was explored. A convenience sample of 399 adults (266 women) completed questionnaires to measure orthorexia nervosa and healthy orthorexia, difficulties in emotion regulation, partnership-related bond, and anxious-depressive-stress symptomatology. The healthy orthorexia subscale was negatively associated with lack of emotional awareness but no other subscale of difficulties in emotion regulation or attachment-related anxiety and avoidance. Orthorexia nervosa scores were positively linked to difficulties in emotion regulation as well as attachment-related anxiety and avoidance. Multiple linear regression indicated non-acceptance of emotional responses and impulse control difficulties to be the strongest predictors for orthorexia nervosa. Both subscales also mediated the effects of attachment style on orthorexia nervosa with anxious-depressive-stress symptomatology moderating some of these effects. Individuals with higher orthorexia nervosa tendencies showed difficulties in emotion regulation, a common feature also of affective and eating disorders. Improvement in understanding the psychological features of orthorexia nervosa can enable a better differentiation from other disorders, advances in the development of treatment approaches and treatment planning, and outlines directions for future research on mechanisms.

## Introduction

Orthorexia nervosa (OrNe) describes the pathological obsession with healthy eating. Although not officially recognized as a mental disorder, obsession over nutrition, severely restricting types of food one eats, and trying to achieve the “perfect” diet can result in severe malnutrition, weight loss, emotional distress and other health issues (1). Up to date, however, the pathological relevance of this behavior is still hotly debated (2). It has been suggested that OrNe should be distinguished from healthy orthorexia (HeOr), defined as a healthy interest in diet and nutrition, and as having a healthy eating identity (3). This proposed two-factor structure has been replicated in follow-up studies showing opposite associations of OrNe and HeOr with disordered eating attitudes, food choices and affective measures (4). In line, orthorexic eating is often accompanied by other disorders, such as eating disorders, obsessive-compulsive as well as affective psychopathology (5–7). These comorbidities substantiate the debate whether OrNe is actually a diagnostic entity that is distinct from established mental disorders (8). At present, this question cannot be readily answered. Some studies clearly identify individuals with symptoms of orthorexia nervosa as a specific group distinct from individuals with eating disorder symptoms or obsessive-compulsive symptoms (9). However, based on commonalities in risk factors, psychological profiles, and clinical symptoms classification of OrNe within the eating disorder spectrum is most popular (10).

According to theoretical models, potentially harmful behaviors share characteristics that facilitate their co-occurrence [e.g., the Problem-Behavior Theory; (11)]. In that regard, difficulties in emotion regulation or the use of maladaptive emotion regulation is a central concept in many mental disorders (12). Emotion regulation encompasses deliberate and automatic processes responsible for monitoring, evaluating, and modifying emotional reactions (13, 14) influencing the quality and intensity of emotions individuals have, and when and how they experience them (15). Thus, difficulties in emotion regulation are related to various disorders' etiology and maintenance (16). Emotion dysregulation is even suggested to constitute a common transdiagnostic factor in mental disorders (17). Emotional dysregulation is also characterized by the inability to tolerate intense, unpleasant emotional states increasing the probability of developing severe mental health problems often associated with problems in impulse control [e.g., self-harm, suicidal ideation; (18)]. In that sense, emotion dysregulation is closely related to eating disorder pathologies. Eating disorder patients often report difficulties in emotion regulation (19, 20) and alexithymia (21). Specifically, two subscales of the difficulties in emotion regulation questionnaire (DERS), the lack of emotional awareness and clarity and low acceptance of emotions show high positive effect sizes in their associations with eating disorders. Moreover, the adaptive emotion regulation strategies re-appraisal and problem-solving show negative associations (22). Two previous studies suggest that emotion regulation might also be one of the factors explaining differences in orthorexic eating tendencies. Vuillier et al. (23) recruited a mainly female UK sample and were the first to relate orthorexic eating tendencies to difficulties in emotion regulations as measured with the Difficulty in Emotion Regulation Scale (DERS). However, after including disordered eating attitudes as a predictor, emotion dysregulation no longer contributed as a statistical predictor of orthorexic eating tendencies. Data from Lebanon confirmed these findings (24). In this study, difficulties in emotion regulation were positively associated with orthorexic tendencies but provided only little additional explained variance beyond current levels of disordered eating. Conclusions from both studies are limited, however, as they both included a version of ORTO-15, a tool criticized for its limited psychometric properties and for not measuring the pathological form of OrNe (25). Still, OrNe is phenomenologically characterized by the expectancy of obtaining positive affective states or mitigating aversive emotional states through eating only healthy food. Hence, negative affective states may predispose to orthorexic eating. Difficulties in emotion regulation may than aggravate the situation and trigger a vicious circle. It could be possible that orthorexic eating is a form of emotion regulation which is used when other strategies do not work or when one does not belief to be capable of using emotion regulation strategies when needed. Orthorexic eating is then used to gain feelings of security by focusing on healthy eating. However, these are only assumptions and need to be tested in (micro-)longitudinal studies. Current diagnostic criteria have partially followed this idea (1, 26) by suggesting emotional consequences of non-adherence to self-imposed nutritional rules as one of three main criteria. Again, this still has to be empirically proven.

Another non-specific vulnerability factor related to emotion regulation and suggested to explain a higher risk for OrNe is insecure attachment. Negative emotions that exceed or challenge the individual emotion regulation competence activate the attachment system (27, 28). In case of secure attachment, individuals seek the physical or psychological closeness of the attachment figures. However, based on relationship experiences of rejection or unpredictable support, already children develop either an insecure-avoidant or an insecure-ambivalent attachment pattern (29). Attachment research in adults distinguishes between insecure-avoidant and insecure-anxious attachment styles. Individuals with an insecure-avoidant attachment style have difficulties to trust their partner, prefer not to depend on them, and avoid being too close. Individuals with an insecure–anxious attachment style feel that their need for closeness in a partnership is not satisfied and fear of being abandoned (30, 31). Attachment theorists assume that attachment avoidance is associated with a deactivating emotion regulation pattern in which the access to negative emotions and the expression and interpersonal regulation is minimized (29, 31). In contrast, the ineffective emotion regulation of individuals with an anxious attachment style is described as hyperactivating and maximizing emotional expression and as inability to change negative emotions. However, empirical evidence also shows that both insecure attachment patterns may only differ in their hierarchy of emotion regulation styles (32). Many models of eating disorders follow the idea that insecure attachment, both anxious or avoidant attachment style, and preoccupied and dismissing representation in close relationships, have important implications in the disorders' development and maintenance (33–35). Insecure attachment patterns in childhood are predictive of emotional eating in adolescence mediated by expressive suppression as emotion regulation strategy (36). Insecure attachment styles have been related to eating disorders accompanied by anxiety, depression, and low self-esteem. Maladaptive emotion regulation is a major mediator between insecure attachment style and symptoms of eating disorders (37). Especially high levels of anxious attachment style predicted eating disorder group membership (38, 39). However, up to date, only one study investigated the contribution of insecure attachment styles to OrNe. Here, both fearful and dismissing attachment styles correlated with higher interest in healthy eating (ORTO-15) but there was no significant additional contribution to explained variance in healthy eating orientation when overweight preoccupation, appearance orientation, and the presence of an eating disorder history were also considered (40).

As noted, OrNe is associated with the experience of increased negative emotions and arousal and even with the increased presence of anxious and depressive symptomatology [for a summary see (2)]. Negative emotional states activate the attachment behavior system and in case of insecure attachment consequently lead to an increased use of maladaptive emotion regulation strategies (29, 32). However, if negative emotions are not elicited, attachment differences in emotion regulation may not be obvious (41). Negative emotional states can also result in an excessive need of self-control but simultaneously in failures in self-regulation and in showing rather impulsive behaviors (e.g., emotional eating) depending on one's fixed mind regarding effective emotion regulation (42). Difficulties in emotion regulation are mediators between insecure attachment style and emotional eating (43). As mentioned above, anxious or avoidant attachment styles are linked to disordered eating behaviors mediated by maladaptive responses to emotions and emotional distress (44). This may especially be the case when experiencing negative emotions like stress, anxiety, or depressive feelings as they activate the attachment system and increase the use of maladaptive emotion regulation. Maladaptive emotion regulation may thus mediate the relationship between attachment style and disordered eating behaviors, including OrNe, with affective symptoms increasing this association. A study looking at this hypothesis in OrNe does not exist yet.

Therefore, this study aimed at investigating two primary goals. First, we aimed to analyze the association of insecure attachment styles with the presence of pathological orthorexic eating (OrNe) separated from the mere interest in healthy eating (HeOr). Second, we explored the mediating role of difficulties in emotion regulation for the association between insecure attachment and OrNe. In these analyses, affective psychopathology is considered to be a moderator expecting that higher affective symptoms reinforce the links. To account for possible gender differences with regard to emotion regulation, attachment, affective psychopathology, and orthorexic behaviors (1, 45), exploratory analyses will quantify the assumed associations separately for men and for women.

## Methods

### Participants

For this study, we used convenience sampling via university mailing lists, social media, posters displayed within the campus, and advertisements in newspapers. Participants had to be aged 16 years and older, and had to clearly assign themselves to the male or female gender. Initially, 434 individuals at least read through the study information (survey page 1). On survey page 2 (consent form), *n* = 3 wanted to stop the survey at this point and *n* = 22 did not give an answer but closed the browser window. No further participant dropped out after providing informed consent. From initially 409 complete data sets, ten sets had to be excluded due to extreme stress levels (open question about major stressor within the last 2 weeks, *n* = 1) and unrealistically fast completion of the survey (i.e., a relative speed index >2, *n* = 9). The final sample consisted of 399 individuals (266 women) aged between 16 and 82 years (median age = 25.00 years).

### Measures

#### Orthorexic Eating

Orthorexic eating was measured by means of the German version of the Teruel Orthorexia Scale [TOS; (3, 46)]. This 17-item tool considers both pathological and healthy aspects of orthorexic eating. Pathological orthorexic eating tendencies are captured through the orthorexia nervosa subscale (TOS-OrNe subscale, eight items, e.g., “I feel guilty when I eat foods that I do not consider healthy”) while the healthy, non-pathological interest in healthy eating is captured through the healthy orthorexia dimension (TOS-HeOr subscale, nine items, e.g. “I feel good when I eat healthy.”). Items are rated on 4-point scales from *0* = “completely disagree” to *3* = “completely agree” with reference to the individual's “general eating behavior”. Internal consistencies were good for both subscales in the present study (*Cronbach's* α TOS-HeOr = 0.84, TOS-OrNe = 0.85).

#### Emotion Regulation

The 36-item Difficulties in Emotion Regulation Scale (47) was employed to analyze different deficits related to suboptimal emotion regulation. We used a German version of the DERS (translation by the authors) for which, like the English-language original, a 6-factor solution was proposed: *non-acceptance of emotional responses* (i.e., non-acceptance of reactions to own distresses, or negative secondary emotional reactions to one's own negative emotions), *difficulties engaging in goal-directed behavior* (i.e., difficulty concentrating and completing tasks in the face of negative emotions), *impulse control difficulties* (i.e., difficulty maintaining control over one's behavior when experiencing negative emotions and the belief that there is little one can do to effectively regulate emotions once upset), *limited access to emotion regulation strategies* (i.e., problems to access strategies for feeling better when distressed), *lack of emotional clarity* (i.e., the extent to which the individual is clear about the emotions they experience), and *lack of emotional awareness* (i.e., tendency to pay only little attention to and to not acknowledge emotions). Answers are given on a 5-point scale with the endpoints “Almost never/0–10% of the time” and “Almost always/90–100% of the time.” Sum scores are created for each subscale. Present good reliability scores confirm previous validation studies (*Cronbach's* α non-acceptance = 0.87, goals = 0.85, impulse = 0.81, strategies = 0.90, clarity = 0.86, awareness = 0.82).

#### Attachment Styles

Attachment style was assessed using a German translation of the Experiences in Close Relationships-Revised [ECR-R; (48, 49)]. This tool allows for the assessment of individual differences in attachment anxiety (i.e., feeling secure or insecure about the availability and responsiveness of romantic partners) and attachment avoidance (i.e., feeling uncomfortable being close to others or depending on others). Items are answered on a 7-point scale where *1* = “strongly disagree” and *7* = “strongly agree”. Mean scores are created. Continuous scores of attachment avoidance and attachment anxiety are used for analyses in concordance with attachment research using this instrument. Internal consistency of both dimensions was good to excellent in the present sample (*Cronbach's* α anxiety = 0.87, avoidance = 0.93).

#### Affective Psychopathology

The Depression-Anxiety-Stress Scales (DASS-21) were used to measure affective symptoms and distress levels (50). Using the 21-item short form, seven items make up each subscale and each item is rated on a 4-point scale from *0* = “Did not apply to me at all” and *3* = “Applied very strongly to me or most of the time.” Answers should be referenced to the last week. Internal consistencies of all subscales were acceptable to good in the present sample (*Cronbach's* α depression = 0.89, anxiety = 0.78, stress = 0.88).

#### Sociodemographics

Besides gender, data on age (years), body mass index (BMI, kg/m^2^), partnerships status (yes/no), current employment (student, employed, other), and regular eating style (vegan, vegetarian, flexitarian, omnivorous) were collected to describe the sample.

### Procedures

Interested individuals were provided with a link to the online survey (platform Soscisurvey). The link was accessible from May to June 2019. Questions were asked in fixed order. Study participation was voluntary, informed consent was attained electronically by clicking the respective statement for approval, and the study was approved by the local ethics committee (University of Wuppertal, reference: MS/BBL 190718). University students received course credit for their participation. All participants completing the questionnaires could additionally participate in a lottery for 5 × 20€ vouchers.

### Statistics

Analyses of this concurrent study were conducted in SPSS v.23 for Mac OS. We standardized all scales using *Z*-scoring. First, zero-order correlations between the orthorexia scales (TOS) and affective psychopathology (DASS-21), emotion regulation difficulties (DERS), attachment styles (ECR-R) were calculated (see Table 1). According to Cohen's criterion, coefficients below 0.29 are considered small, values between 0.30 and 0.49 are interpreted as medium correlation, and values above 0.50 represent strong correlations. Coefficients below 0.20 were considered negligible and are thus not interpreted. We additionally explored the possible confounding effects of age. First, we correlated age with variables under study. Except one meaningful negative correlation with insecure-anxious attachment, there were no significant correlations. Second, we computed partial correlations controlling for age. In addition, we repeated all analyses excluding participants aged >60 years. Coefficients hardly changed indicating negligible influence of this factor (Supplementary Material). Moreover, differences in these associations between women and men were examined in gender-separate analyses complementing the total sample analyses. For the comparison of the two correlations Fisher's *z* transformation was used (Supplementary Material). Overall gender differences could not be verified. Following analyses were therefore conducted without both variables. Second, stepwise multiple regressions evaluated the predictive role of emotion regulation and attachment relative to depressive-anxious-stress symptomatology and healthy/pathological orthorexic eating. Third, a moderated mediation analysis was conducted using the PROCESS macro (version 3.5) to analyze how the independent variables (insecure-anxious attachment, insecure-avoidant attachment) affect the dependent variable (pathological orthorexic eating) through multiple mediators (emotion regulation) and moderators (symptoms of depression, anxiety, stress). Thus, the effects of the predictors (ECR-R subscales) on the mediator variables (DERS subscales) and the effects of mediator variables on the dependent variable (TOS-OrNe) were examined while considering the moderating effects of affective symptoms (DASS-21 subscales). The direct (c') and conditional indirect effects were estimated for each predictor and moderator in an independent model (model 58). Indirect effects of mediators were examined by bootstrapping (5,000 bootstraps). In case of full mediation, c' = 0. Results for the conditional indirect effects are reported as 95% confidence intervals with intervals that do not contain zero were considered significant (51). A heteroscedasticity consistent standard error and covariance matrix estimator was used because the existence of heteroskedasticity could not be excluded from the scatterplot of residuals. *P*-values < 0.05 were considered significant. Controlling for multiple testing, Bonferroni correction was applied with *p*_*corrected*_ < 0.05/8 and 0.05/6 for the regression (six DERS and two ECR-R subscales) and mediation (two ECR-R and three DASS-21 subscales) models, respectively.

**Table 1:** Pearson correlations of variables under study for the total group.

**Variable**	***M***	***SD***	**Correlation**											
			**(1)**	**(2)**	**(3)**	**(4)**	**(5)**	**(6)**	**(7)**	**(8)**	**(9)**	**(10)**	**(11)**	**(12)**
(1) TOS HeOr	12.10	5.09												
(2) TOS OrNe	3.34	3.77	0.472^***^											
(3) DASS-21 Depression	3.07	3.77	−0.027	0.341^***^										
(4) DASS-21 Anxiety	1.76	2.59	0.041	0.319^***^	0.672^***^									
(5) DASS-21 Stress	4.46	4.11	0.039	0.358^***^	0.757^***^	0.676^***^								
(6) DERS Non-acceptance	11.11	4.81	0.089	0.479^***^	0.495^***^	0.431^***^	0.546^***^							
(7) DERS Goals	12.31	4.25	−0.014	0.278^***^	0.445^***^	0.407^***^	0.524^***^	0.523^***^						
(8) DERS Impulse	10.86	3.91	0.069	0.444^***^	0.481^***^	0.401^***^	0.609^***^	0.672^***^	0.617^***^					
(9) DERS Strategies	15.82	6.39	−0.007	0.406^***^	0.674^***^	0.564^***^	0.636^***^	0.725^***^	0.682^***^	0.730^***^				
(10) DERS Clarity	9.58	3.70	−0.128^*^	0.280^***^	0.434^***^	0.341^***^	0.396^***^	0.500^***^	0.328^***^	0.466^***^	0.542^***^			
(11) DERS Awareness	15.00	4.52	−0.208^***^	0.121^*^	0.245^***^	0.163^**^	0.190^***^	0.287^***^	0.115^*^	0.292^***^	0.315^***^	0.618^***^		
(12) ECR-R Anxiety	2.83	0.98	−0.108^*^	0.220^***^	0.393^***^	0.373^***^	0.388^***^	0.369^***^	0.378^***^	0.334^***^	0.467^***^	0.361^***^	0.188^***^	
(13) ECR-R Avoidance	2.65	1.12	−0.087	0.181^***^	0.330^***^	0.239^***^	0.218^***^	0.278^***^	0.137^**^	0.263^***^	0.309^**^	0.394^***^	0.422^***^	0.435^***^

*TOS, Teruel Orthorexia Scale; HeOr, healthy orthorexia; OrNe, Orthorexia nervosa; DASS-21, Depression-Anxiety-Stress Scales; DERS, Difficulties in Emotion Regulation Scale; ECR-R, Experiences in Close Relationships – Revised*.

*** *p < 0.001*,

** *p < 0.01*,

* *p < 0.05. r > 0.2*.

## Results

Participants were between 16 and 82 years of age, with a mean age of 30.53 ± 12.90 years, and with *n* = 16 (4.0 %) individuals being older than 60 years. The BMI ranged between 16.20 and 40.56 kg/m^2^, with a mean of 22.89 ± 3.64 kg/m^2^. Four individuals had a BMI < 17 kg/m^2^ (three women, one men) and *n* = 19 were overweight (BMI > 30 kg/m^2^, 11 women, eight men). About 2/3 of the sample reported to be in a solid partnership (*n* = 256, 64.2%). About half of the sample were students (*n* = 212, 53.1%) and one third were employed (*n* = 141, 35.3%). Some kind of restriction in their regular diet was reported by 42.6% of the sample (5.3% vegan, 17.5% vegetarian, 19.8% flexitarian, 57.4% omnivore).

Pearson correlations of all variables are reported in Table 1. All emotion regulation subscales of the DERS correlated positively with TOS-OrNe (orthorexia nervosa), though with small to moderate effects. Only the subscale “lack of emotional awareness” correlated below r = 0.20. Attachment anxiety was significantly positively associated with TOS-OrNe, but attachment avoidance was not. In contrast, the TOS-HeOr subscale for healthy orthorexia was uncorrelated to all DERS and ECR-R subscales except with the DERS scale “lack of emotional awareness” which showed a small negative association. Gender differences were hardly shown. Only the associations of DASS depression and attachment anxiety with TOS-HeOr (stronger negative correlation in men), and of DASS stress and DERS lack of emotional awareness with TOS-OrNe (stronger positive correlation in women) were significantly different between men and women. As already noted above, variables were uncorrelated to age (except insecure-anxious attachment).

Table 2 shows results from the multiple regression analyses examining the statistical prediction of psychopathological symptoms assessed in the DASS and symptoms of orthorexia from insecure attachment styles (ECR-R) and emotion regulation difficulties (DERS). The model explained 47% of depressive symptoms assessed in the DASS, *R*^2^ adjusted = 0.47, *F*_(2,396)_ = 176.4, *p* < 0.001, with limited access to strategies and attachment avoidance being significant predictors. Individuals with more limited access to emotion regulation strategies and scoring higher in avoidant attachment were more likely to report higher scores in depressive symptoms. The same predictors significantly explained for symptoms of anxiety assessed in the DASS (explained variance 33%, *R*^2^ adjusted = 0.33, *F*_(2,396)_ = 99.1, *p* < 0.001). For stress, the model explained 46% variance, *R*^2^ adjusted = 0.46, *F*_(3,395)_ = 112.2, *p* < 0.001. Here, the DERS subscales limited access to strategies and impulse control difficulties as well as attachment anxiety were significant predictors. In the TOS-HeOr model, predictors explained 8% variance, *R*^2^ adjusted = 0.08, *F*_(3,395)_ = 12.1, *p* < 0.001. The DERS subscales lack of emotional awareness and non-acceptance and also attachment anxiety were significant predictors. Individuals with less impaired awareness of their feelings, higher scores in non-acceptance of emotions and low scores in attachment anxiety reported higher healthy orthorexic eating behaviors. Finally, the model explained 26% of variance in TOS-OrNe, *R*^2^ adjusted = 0.25, *F*_(2,396)_ = 68.3, *p* < 0.001. The DERS subscales non-acceptance and impulse control difficulties were significant predictors. Higher pathological orthorexic eating was reported by individuals scoring higher on both scales.

**Table 2:** Linear multiple regressions of difficulties in emotion regulation and attachment style on symptoms of depression, anxiety, stress, and orthorexic eating.

	**β**	***t***	***p***
**Depression (*****R*** **= 0.69, *R*^2^ = 0.47, adjusted *R*^2^ = 0.47**, ***p*** **< 0.001)**			
DERS Limited strategies	0.633	16.47	<0.001
ECR-R Avoidance	0.134	3.50	0.001
**Anxiety (*****R*** **= 0.58, *R*^2^ = 0.33, adjusted *R*^2^ = 0.33**, ***p*** **< 0.001)**			
DERS Limited strategies	0.499	10.75	<0.001
ECR-R Avoidance	0.140	3.02	0.003
**Stress (*****R*** **= 0.68, *R*^2^ = 0.46, adjusted *R*^2^ = 0.46**, ***p*** **< 0.001)**			
DERS Limited strategies	0.353	6.13	<0.001
DERS Impulse control difficulties	0.311	5.75	<0.001
ECR-R Anxiety	0.119	2.84	0.005
**Healthy orthorexia (*****R*** **= 0.29, *R*^2^ = 0.08, adjusted *R*^2^ = 0.08**, ***p*** **< 0.001)**			
DERS Lack of awareness	−0.242	−4.80	<0.001
DERS Non-acceptance	0.210	3.94	<0.001
ECR-R Anxiety	−0.140	−2.70	0.007^#^
**Orthorexia nervosa (*****R*** **= 0.51, *R*^2^ = 0.26, adjusted *R*^2^ = 0.25**, ***p*** **< 0.001)**			
DERS Non-acceptance	0.329	5.62	<0.001
DERS Impulse control difficulties	0.223	3.82	<0.001

*DERS, Difficulties in Emotion Regulation Scale; ECR-R, Experiences in Close Relationships – Revised*.

# *Not significant after multiple testing correction*.

**Figure 1:**
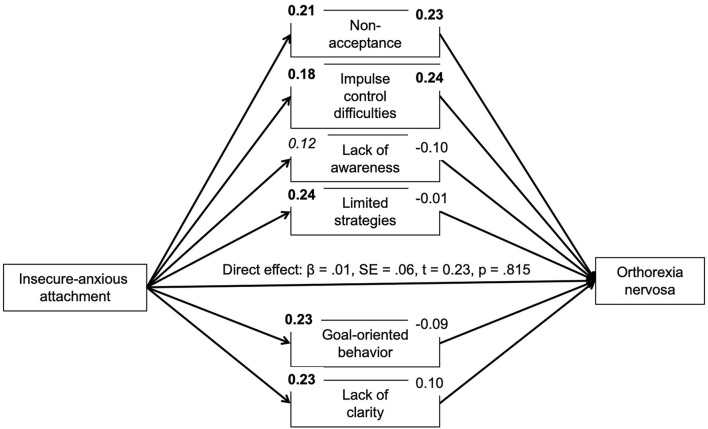
Moderated mediation results for the relationship between anxious-insecure attachment and pathological orthorexic eating as mediated by difficulties in emotion regulation (moderator: depressive symptoms). Bold *p* < 0.008, italic *p* < 0.05.

**Figure 2:**
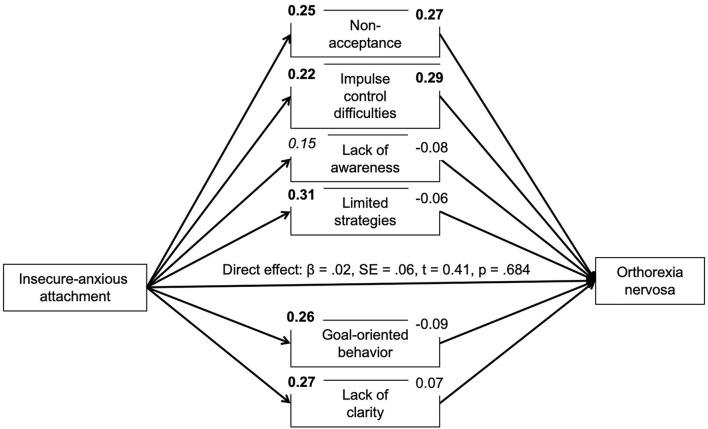
Moderated mediation results for the relationship between anxious-insecure attachment and pathological orthorexic eating as mediated by difficulties in emotion regulation (moderator: anxiety symptoms). Bold *p* < 0.008, italic *p* < 0.05.

**Figure 3:**
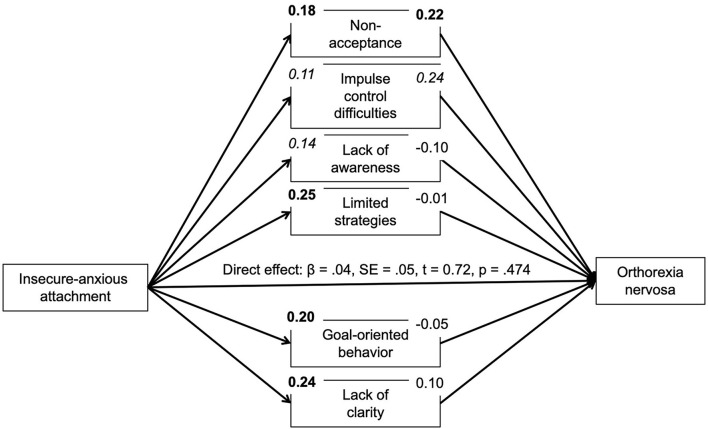
Moderated mediation results for the relationship between anxious-insecure attachment and pathological orthorexic eating as mediated by difficulties in emotion regulation (moderator: stress symptoms). Bold *p* < 0.008, italic *p* < 0.05.

**Figure 4:**
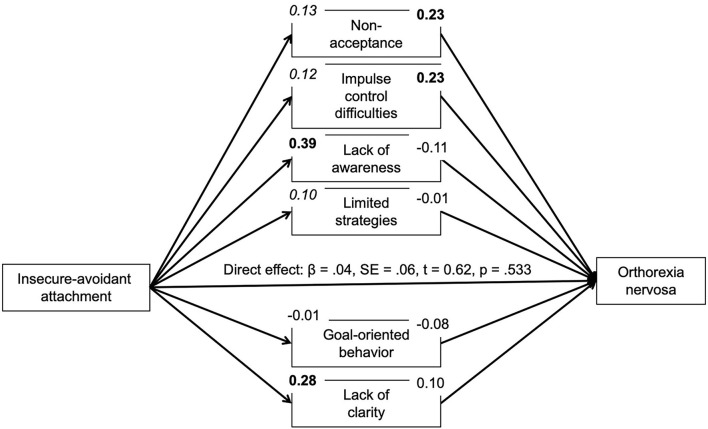
Moderated mediation results for the relationship between avoidant attachment and pathological orthorexic eating as mediated by difficulties in emotion regulation (moderator: depressive symptoms). Bold *p* < 0.008, italic *p* < 0.05.

**Figure 5:**
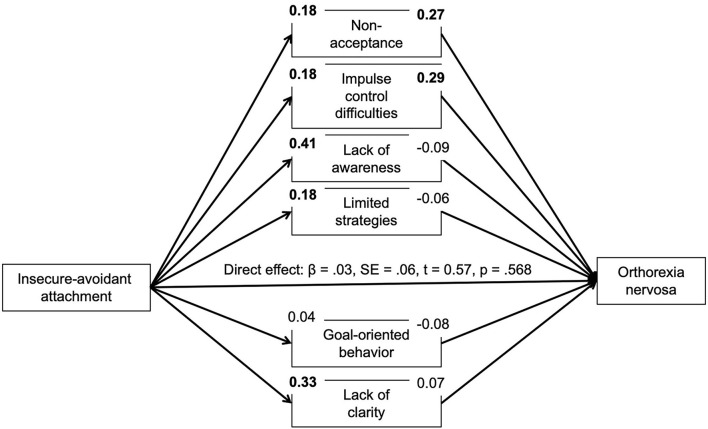
Moderated mediation results for the relationship between avoidant attachment and pathological orthorexic eating as mediated by difficulties in emotion regulation (moderator: anxiety symptoms). Bold *p* < 0.008, italic *p* < 0.05.

**Figure 6:**
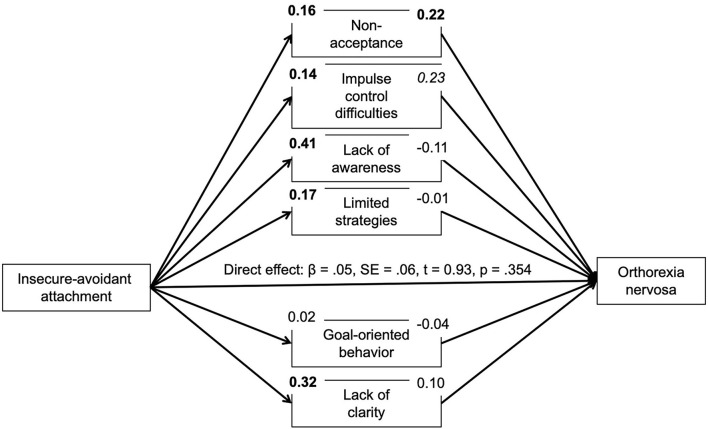
Moderated mediation results for the relationship between avoidant attachment and pathological orthorexic eating as mediated by difficulties in emotion regulation (moderator: stress symptoms). Bold *p* < 0.008, italic *p* < 0.05.

Based on correlational analyses, the proposed mediating role of difficulties in emotion regulation for the association between insecure attachment and pathological orthorexic eating moderated by affective psychopathology was examined using a moderated mediation model, controlling for the effects of gender, age, and BMI. In regard to insecure-anxious attachment, the a-path (predictor on mediator effect) was always significant or nearly significant. The b-path was only significant for a few mediators. Namely, an indirect effect of non-acceptance and impulse control difficulties was found. The conditional indirect effect of non-acceptance appeared stronger at mean [*b* = 0.04, 95% *BC CI* (0.01–0.09)] and high levels {+SD, [*b* = 0.07, 95% *BC CI* (0.02–0.13)]} of depressive symptoms compared to low (–SD) levels. The conditional indirect effect of impulse control difficulties appeared stronger at low {–SD, [*b* = 0.05, 95% *BC CI* (0.01–0.12)]} and mean levels [*b* = 0.04, 95% *BC CI* (0.01–0.09)] of depressive symptoms compared to high (+SD) levels. While there was no moderating effect of anxiety symptoms, the conditional indirect effect of non-acceptance appeared stronger at high levels of stress {+SD, [*b* = 0.07, 95% *BC CI* (0.02–0.13)]} compared to low (–SD) and mean levels. In regard to attachment avoidance, the model including depressive symptoms as moderator showed no indirect effects. By contrast, the model including anxiety symptoms showed indirect effects of non-acceptance and impulse control difficulties on symptoms of orthorexia nervosa. The indirect effect of non-acceptance was not moderated by anxiety symptoms. The conditional indirect effect of impulse control difficulties, however, appeared stronger at low {–*SD*, [*b* = 0.06, 95% *BC CI* (0.01–0.12)]} and mean levels [*b* = 0.06, 95% *BC CI* (0.01–0.11)] of anxious symptoms compared to high (+*SD*) levels. The model including the moderator stress showed an indirect effect of non-acceptance which appeared stronger at high levels of stress {+*SD*, [*b* = 0.07, 95% *BC CI* (0.02–0.13)]} compared to low (–*SD*) and mean levels (see Figures 1–6).

## Discussion

The aim of the study was to examine the associations of emotion regulation difficulties and insecure attachment styles with signs of healthy and pathological orthorexic behaviors. The findings of this study with a convenience sample showed that specific dimensions of emotion regulation difficulties correlated with pathological orthorexic behaviors while there was no relation to the healthy aspects of orthorexic eating. Overall, gender differences in these associations were rare. Attachment style played a role for women, less so for men. Mediation models implicated that non-acceptance of emotions and impulse control difficulties fully mediated the relationship between attachment style and pathological orthorexic behaviors. Current affective symptoms partially moderated this association.

### Emotion Dysregulation and Orthorexia Nervosa

In case of emotion regulation, the positive associations between all domains of difficulties in emotion regulation and symptoms of orthorexia nervosa were in accordance with similar results obtained from eating disordered populations who also score higher on emotion regulation difficulties or show maladaptive emotion regulation (19, 20). Previous studies of anorexia and bulimia nervosa have found lower distress tolerance, more impulsive behaviors when distressed, deficits in the flexible use of adaptive and situationally appropriate strategies as well as deficits in emotion understanding (52–54). The present study did only find a small association between OrNe and the subscale of the DERS assessing emotional self-awareness deficits. This is in contrast to numerous studies on alexithymia in Anorexia nervosa and Bulimia nervosa (55). Importantly, the effect sizes were only small (for problems with goal-directed behavior and lack of emotional clarity) to medium (for non-acceptance of emotions, impulse control difficulties and limited access to emotion regulation strategies). This, however, is comparable to previous studies on pathological healthful eating. One study showed DERS problems with clarity of emotions to be unrelated to preoccupation with healthy food, whereas the difficulties in impulse control and access to emotion regulation strategies dimension of the DERS correlated to a moderate degree (23). The data from the second study in this area did not show stronger associations [all *r* < 0.3; (24)]. This may suggest that there are small linear effects of difficulties in emotion regulation, when studying a sample with few participants showing orthorexic behaviors. Regression analyses showed that emotion dysregulation predicted orthorexic behaviors and affective symptomatology, limited access to strategies and impulse control difficulties in particular. This supports previous theories that consider emotion dysregulation a transdiagnostic factor playing a key role in various psychiatric disorders (17). On the other hand, previous theories that consider pathological eating behaviors and OrNe as a way of distracting from negative affect and a consequence of self-control failures also fit the results (1, 26). Two dimensions that stood out in the mediation analyses were non-acceptance and impulse control difficulties. Both of them mediated the predictive effects of insecure attachment styles on OrNe. Non-acceptance measures negative secondary emotional reactions to one's own negative emotions, individuals react to their emotions with discomfort or shame. Failures to keep control over one's feelings and behaviors define the impulse dimension. Both facets are defining characteristics of other impulse control disorders and addictive behaviors (42). Whether orthorexic eating may serve as a way of alleviating depressive, anxious and stress symptoms or whether affective symptoms aggravate orthorexic eating leaving individuals in a vicious cycle has neither been examined using experimental nor naturalistic study designs. In regard to the latter, ecological momentary assessment studies would allow to examine the bi-directional link between negative affect and OrNe. Studies in eating disorders indicate that negative affect precedes binge and purging episodes but challenge reductions in negative affect after these compensatory behaviors (56, 57). Initial experimental studies showing weaknesses in emotional control, cognitive inflexibility and attentional bias toward healthy food (58, 59) support this alleviation assumption in OrNe. Studies using mood induction in relation to food cue reactivity are still missing, additional research is thus needed. Overall, even if the correlations are not as strong as in the case of already established clinical diagnoses of eating disorders, the findings are consistent. Emotion dysregulation appeared as a common predictor of orthorexic eating and affective psychopathology. Exploring whether the treatment of emotion regulation difficulties may be beneficial for reducing this behavior and comorbid symptoms seems a fruitful future endeavor.

### Attachment and Orthorexia Nervosa

The results showed that anxious attachment was linked to orthorexic eating, but there was only a small association with attachment avoidance in this sample. Importantly, this positive association was driven mainly by the female sample. These results corroborate results of the only other study examining associations of attachment with pathological healthful eating. In this previous report, the extent of healthful eating was weakly associated with both fearful and dismissing/avoidant attachment style (40). However, history of eating disorders and appearance related aspects reduced the significant association. The association's direction is thus consistent with observed links in eating disorders (38, 47, 60). Especially patients with anorexia nervosa show problems reflecting on their behavior in relationships. Moreover, insecure attachment style negatively affects the therapeutic process and the treatment success (61). Mediation analyses showed that attachment did play a role in explaining OrNe scores through maladaptive emotion regulation. The development of secure attachment is an important developmental task (41, 62), subsequent expression of emotion regulation strategies may than confer the risk for maladjustment, specifically developing problematic eating (35). Present results support this assumption, in particular for non-acceptance of emotional responses and impulse control difficulties when distressed. Moderated mediated regressions showed the assumed reinforcing effects of depressive and stress symptoms for the mediating effects of non-acceptance for insecure-anxious attachment style. Results are different for insecure-avoidant attachment style. For the indirect path from avoidant attachment to symptoms of orthorexia nervosa mediated by impulse control difficulties, the results indicated a more pronounced mediation at low and mean depressive and anxious symptoms. Avoidant attachment style is characterized by an increased tendency to control and deactivate feeling and expressing negative emotions or needs, especially in social contexts (29, 32). Difficulties in controlling own impulses and feeling out of control as assessed in this DERS scale counteract this avoidance tendency. Thus, already low to moderate anxiety symptoms may activate the pathological orthorexic symptoms of feeling guilty when losing control and eating unhealthy food. High intensity of anxiety symptoms may no longer increase this link. However, this is the first study examining such a moderated mediation. Other studies mainly examined either whether negative affect is a reinforcer of emotional or pathological eating (63) or whether insecurely attached individuals have an increased risk for developing symptoms of eating disorder (63, 64). However, the associations between attachment avoidance and eating disorder symptoms associated with impulse control (e.g., binge eating, emotional eating) are clearly lower than the associations with insecure anxious attachment (64). One additional perspective might be that in the case of above-average depression/anxiety levels, there is simply a lack of resources to pursue a healthy lifestyle. Since the mean values of affective symptoms were comparably low in our rather healthy sample, the results must not be overinterpreted. Micro-longitudinal study approaches are warranted to investigate this further.

### Differentiating Healthy Orthorexia Behavior From Orthorexia Nervosa

We found differential effects for healthy orthorexic behaviors. Here, the TOS-HeOr subscale was unrelated to all dimensions of attachment and emotion dysregulation except lack of emotional awareness. Individuals who pay only little attention to and do not acknowledge emotions showed lower healthy orthorexic behaviors. Regression analysis confirmed this and showed anxious attachment to be also negatively related to HeOr. Additionally, non-acceptance was a relevant predictor but in contrast to our expectations with a positive coefficient. This is surprising given the missing correlation between this dimension and HeOr in the bivariate analysis. Furthermore, this contradicts previous studies showing healthy dietary patterns, e.g., Mediterranean diet, to be associated with fewer difficulties in emotion regulation (65). In general, individuals with better emotion regulation capabilities are more likely to engage in healthy behaviors (66). It may be possible that other important variables may moderate the association between emotion dysregulation and HeOr. In particular, motivation and consciousness of individual goals may be relevant. For instance, non-accepting individuals who show achievement-oriented goals (e.g., health and fitness) rather than emotion-oriented goals may display a higher interest in healthy eating in the sense of compensatory behavior. Such moderating relationships have to be explored in future studies.

### Gender Effects

Examining gender differences was not a main aim of this study. However, given our previous knowledge of gender differences in orthorexic eating and underlying developmental mechanisms (1), gender-separate correlation analyses were conducted. In fact, significant gender differences were only shown for anxious attachment style with HeOr (only significant in men) and lack of emotional awareness with OrNe (only significant in women). Anxious attachment style is quite prominent in females compared to males and the associated trend to a more hyperactivating emotion regulation style (29, 31). This might be due to the diverse functions that healthy and orthorexic eating may serve differentially between genders. Whether these diverging associations also suggest differential developmental mechanisms of orthorexic eating in men and women is difficult to conclude from the present data and the sample studied here. Unequal groups sizes and potential selection bias may undermine generalizability of findings and thus require replication.

### Limitations

Findings should be interpreted in light of a number of limitations. First, the age range of our sample was rather wide which could generate important variability and bias due age-specific influences on attitudes or symptoms regarding healthy eating, depending on changing health status. Additional analyses neither indicated meaningful correlations between age and study variables (except a negative correlation with insecure-anxious attachment style) nor did results change when excluding older participants. Findings thus appear to be robust and do not meaningfully covary with age. However, age differences and associated health status need to be considered more closely. As already noted, the sample included a high percentage of female participants and convenience sampling was applied leaving generalizability unclear. In addition, the study did not specifically control for eating disorders. Thus, this study cannot address pathological eating as a possible contributor to the findings and what orthorexic eating contributes beyond eating disorder attitudes. Finally, the study design only allows the analysis of concurrent associations. The examined mediational processes need to be replicated longitudinally controlling for current eating disorder problems in order to examine more causal mechanisms.

## Conclusion

In conclusion, the results of the present study suggest that individuals with higher pathological orthorexic eating have increased difficulties in regulating their emotions implying that this may serve as a risk factor for Orthorexia nervosa. In addition, they also showed a more insecure attachment pattern. Therefore, our findings show parallels to findings for several affective and eating disorders (35). Accordingly, the relationship between insecure attachment and pathological orthorexic eating tendencies was mediated by emotion dysregulation. Moreover, symptoms of affective psychopathology partly moderated these mediations. The non-pathological interest in healthy dieting was not related to emotion dysregulation and insecure attachment, thus confirming assumptions of a bi-dimensional structure of orthorexic tendencies.

Findings imply that emotion dysregulation and attachment style are important determinants of mental health in Orthorexia nervosa. Whether both factors should be considered as potential vulnerability factors or diagnostic criteria, how they fit into models of development, and whether their consideration would advance approaches to treatment and counseling for orthorexic eating behavior are important future questions. The differentiated findings regarding pathological and healthy orthorexic eating can also be helpful for the diagnosis of Orthorexia nervosa, for example, when clarifying whether the behavior is pathological and clinically relevant or merely a healthy interest in nutrition.

For future studies and to solidify the results, in addition to the use of self-report measurements, the use of other diagnostic tools such as clinical interviews would be desirable. Concerning the acquisition of emotion dysregulation, the use of the ecological momentary assessment (EMA) method in future studies will enrich our understanding of emotion regulation processes in healthy and pathological orthorexic behaviors.

## Data Availability Statement

The raw data supporting the conclusions of this article will be made available by the authors, without undue reservation.

## Ethics Statement

The studies involving human participants were reviewed and approved by University of Wuppertal, reference: MS/BBL 190718. The patients/participants provided their written informed consent to participate in this study.

## Author Contributions

JS and PZ conceived and designed the study. JS collected the data and performed the analyses. JS and SN wrote the first article draft. HW and PZ revised the article. All authors discussed the results and contributed to the final manuscript and have approved the final article.

## Funding

The article processing charge was partially funded by the Baden-Württemberg Ministry of Science, Research and Art and the University of Freiburg in the funding programme Open Access Publishing.

## Conflict of Interest

The authors declare that the research was conducted in the absence of any commercial or financial relationships that could be construed as a potential conflict of interest.

## Publisher's Note

All claims expressed in this article are solely those of the authors and do not necessarily represent those of their affiliated organizations, or those of the publisher, the editors and the reviewers. Any product that may be evaluated in this article, or claim that may be made by its manufacturer, is not guaranteed or endorsed by the publisher.
