# Supplementary material for: Psychological Correlates of Excessive Healthy and Orthorexic Eating: Emotion Regulation, Attachment, and Anxious-Depressive-Stress Symptomatology
Source: Front Nutr. 2022 Mar 9;9:817047. doi: 10.3389/fnut.2022.817047 (PMC8959669; doi:10.3389/fnut.2022.817047)
Supplement: Supplementary file 1 [file Data_Sheet_1.docx]

**Supplementary material for** *Psychological correlates of excessive healthy and orthorexic eating: emotion regulation, attachment and anxious-depressive-stress symptomatology* by Jana Strahler, Hanna Wachten, Shanna Neuhofer, Peter Zimmermann

**Table of contents**

**Supplementary Table 1.** Pearson correlations of variables under study in the male sample.

**Supplementary Table 2.** Pearson correlations of variables under study in the female sample.

**Supplementary Table 3**. Pearson correlations of variables under study, for the total group and additionally separated by gender.

**Supplementary Table 4.** Pearson correlations of age with variables under study.

**Supplementary Table 5**. Pearson correlations of variables under study for the subsample aged ≤ 60 years (*N* = 383).

**Supplementary Table 6.** Pearson correlations of variables under study in the male subsample aged ≤ 60 years (*N* = 120).

**Supplementary Table 7.** Pearson correlations of variables under study in the female subsample aged ≤ 60 years (*N* = 263).

**Supplementary Table 8**. Partial correlations, controlling for age, between study variables for the total sample.

**Supplementary Table 9.** Partial correlations, controlling for age, between study variables for the male sample.

**Supplementary Table 10.** Partial correlations, controlling for age, between study variables for the female sample.

**Supplementary Table 1.** Pearson correlations of variables under study in the male sample.

| **Variable** | ***M*** | ***SD*** | **correlation** | | | | | | | | | | | |
| --- | --- | --- | --- | --- | --- | --- | --- | --- | --- | --- | --- | --- | --- | --- |
|  |  |  | (1) | (2) | (3) | (4) | (5) | (6) | (7) | (8) | (9) | (10) | (11) | (12) |
| (1) TOS HeOr | 11.66 | 5.31 |  |  |  |  |  |  |  |  |  |  |  |  |
| (2) TOS OrNe | 3.06 | 3.33 | .480^***^ |  |  |  |  |  |  |  |  |  |  |  |
| (3) DASS-21 Depression | 2.55 | 3.37 | -.245^**^ | .129 |  |  |  |  |  |  |  |  |  |  |
| (4) DASS-21 Anxiety | 1.27 | 1.99 | -.075 | .202^*^ | .662^***^ |  |  |  |  |  |  |  |  |  |
| (5) DASS-21 Stress | 3.31 | 3.68 | -.116 | .146 | .737^***^ | .730^***^ |  |  |  |  |  |  |  |  |
| (6) DERS Non-acceptance | 9.84 | 4.14 | .141 | .511^***^ | .428^***^ | .331^***^ | .455^***^ |  |  |  |  |  |  |  |
| (7) DERS Goals | 11.77 | 4.31 | -.033 | .328^***^ | .423^***^ | .386^***^ | .476^***^ | .547^***^ |  |  |  |  |  |  |
| (8) DERS Impulse | 10.06 | 3.36 | -.014 | .481^***^ | .348^***^ | .315^***^ | .487^***^ | .683^***^ | .582^***^ |  |  |  |  |  |
| (9) DERS Strategies | 14.41 | 5.86 | -.076 | .378^***^ | .623^***^ | .479^***^ | .559^***^ | .758^***^ | .690^***^ | .718^***^ |  |  |  |  |
| (10) DERS Clarity | 9.45 | 3.74 | -.216^*^ | .209^*^ | .416^***^ | .301^***^ | .393^***^ | .471^***^ | .314^***^ | .460^***^ | .519^***^ |  |  |  |
| (11) DERS Awareness | 15.38 | 4.80 | -.300^***^ | -.065 | .250^***^ | .128 | .174^*^ | .097 | .008 | .183^***^ | .191^*^ | .614^***^ |  |  |
| (12) ECR-R Anxiety | 2.76 | 0.98 | -.234^**^ | .133 | .408^***^ | .385^***^ | .403^***^ | .363^***^ | .482^***^ | .324^***^ | .449^***^ | .279^***^ | .037 |  |
| (13) ECR-R Avoidance | 2.76 | 1.14 | -.169 | .094 | .348^***^ | .161 | .231^**^ | .190^*^ | .106 | .273^***^ | .185^*^ | .294^***^ | .409^***^ | .265^**^ |

*TOS, Teruel Orthorexia Scale; HeOr, healthy orthorexia; OrNe, Orthorexia nervosa; DASS-21, Depression-Anxiety-Stress Scales; DERS, Difficulties in Emotion Regulation Scale; ECR-R, Experiences in Close Relationships – Revised. ***p<.001, **p<.01, *p<.05. r>0.2*

**Supplementary Table 2.** Pearson correlations of variables under study in the female sample.

| **Variable** | ***M*** | ***SD*** | **correlation** | | | | | | | | | | | |
| --- | --- | --- | --- | --- | --- | --- | --- | --- | --- | --- | --- | --- | --- | --- |
|  |  |  | (1) | (2) | (3) | (4) | (5) | (6) | (7) | (8) | (9) | (10) | (11) | (12) |
| (1) TOS HeOr | 12.31 | 4.98 |  |  |  |  |  |  |  |  |  |  |  |  |
| (2) TOS OrNe | 3.47 | 3.96 | .469^***^ |  |  |  |  |  |  |  |  |  |  |  |
| (3) DASS21 Depression | 3.32 | 3.89 | .064 | .414^***^ |  |  |  |  |  |  |  |  |  |  |
| (4) DASS21 Anxiety | 2.01 | 2.82 | .074 | .350^***^ | .674^***^ |  |  |  |  |  |  |  |  |  |
| (5) DASS21 Stress | 5.04 | 4.20 | .118 | .432^***^ | .763^***^ | .654^***^ |  |  |  |  |  |  |  |  |
| (6) DERS Non-acceptance | 11.74 | 5.00 | .053 | .467^***^ | .509^***^ | .444^***^ | .555^***^ |  |  |  |  |  |  |  |
| (7) DERS Goals | 12.58 | 4.21 | -.012 | .254^***^ | .450^***^ | .413^***^ | .540^***^ | .508^***^ |  |  |  |  |  |  |
| (8) DERS Impulse | 11.26 | 4.10 | .095 | .428^***^ | .518^***^ | .411^***^ | .638^***^ | .656^***^ | .631^***^ |  |  |  |  |  |
| (9) DERS Strategies | 16.53 | 6.53 | .012 | .413^***^ | .689^***^ | .583^***^ | .651^***^ | .703^***^ | .676^***^ | .726^***^ |  |  |  |  |
| (10) DERS Clarity | 9.65 | 3.69 | -.083 | .310^***^ | .443^***^ | .362^***^ | .403^***^ | .522^***^ | .334^***^ | .474^***^ | .557^***^ |  |  |  |
| (11) DERS Awareness | 14.81 | 4.37 | -.150^*^ | .212^***^ | .256^***^ | .195^**^ | .223^***^ | .402^***^ | .185^**^ | .363^***^ | .399^***^ | .624^***^ |  |  |
| (12) ECR-R Anxiety | 2.87 | 0.98 | -.047 | .254^***^ | .384^***^ | .370^***^ | .378^***^ | .369^***^ | .320^***^ | .335^***^ | .473^***^ | .401^***^ | .277^***^ |  |
| (13) ECR-R Avoidance | 2.59 | 1.11 | -.036 | .226^***^ | .337^***^ | .289^***^ | .240^***^ | .344^***^ | .165^**^ | .281^***^ | .390^***^ | .450^***^ | .426^***^ | .530^**^ |

*TOS, Teruel Orthorexia Scale; HeOr, healthy orthorexia; OrNe, Orthorexia nervosa; DASS-21, Depression-Anxiety-Stress Scales; DERS, Difficulties in Emotion Regulation Scale; ECR-R, Experiences in Close Relationships – Revised. ***p<.001, **p<.01, *p<.05. r>0.2*

**Supplementary Table 3**. Pearson correlations of variables under study, for the total group and additionally separated by gender.

| **Variable** | **TOS-HeOr** | | **TOS-OrNe** |  |
| --- | --- | --- | --- | --- |
|  | **Fisher’s *z*** | **Fisher’s *z*** | |  |
| DASS-21 Depression | **2.930, *p* = .002** | **2.032, *p* = .021** | |  |
| DASS-21 Anxiety | 1.392, *p* = .082 | 1.498, *p* = .067 | |  |
| DASS-21 Stress | **2.193, *p* = .014** | **2.941, *p* = .002** | |  |
| DERS Non-acceptance | -0.829, *p* = .204 | -0.432, *p* = .333 | |  |
| DERS Goals | 0.196, *p* = .422 | -0.755, *p* = .225 | |  |
| DERS Impulse | 1.019., *p* = .154 | -0.623, *p* = .267 | |  |
| DERS Strategies | 0.822, *p* = .205 | 0.387, *p* = .349 | |  |
| DERS Clarity | 1.271, *p* = .102 | 1.011, *p* = .156 | |  |
| DERS Awareness | 1.477, *p* = .070 | **2.615, *p* = .004** | |  |
| ECR-R Anxiety | **1.785, *p* = .037** | 1.174, *p* = .120 | |  |
| ECR-R Avoidance | 1.256, *p* = .105 | 1.266, *p* = .103 | |  |
| *TOS, Teruel Orthorexia Scale; HeOr, healthy orthorexia; OrNe, Orthorexia nervosa; DASS-21, Depression-Anxiety-Stress Scales; DERS, Difficulties in Emotion Regulation Scale; ECR-R, Experiences in Close Relationships – Revised.* | | | | |

**Supplementary Table 4**. Pearson correlations of age with variables under study.

| **Variable** | **correlation with age** | | |
| --- | --- | --- | --- |
|  | total sample (*N* = 383) | female sample (*N* = 263) | male sample (*N* = 120) |
| (1) TOS HeOr | -.010 | -.048 | .080 |
| (2) TOS OrNe | -.111^*^ | -.106 | -.114 |
| (3) DASS-21 Depression | -.101^*^ | -.037 | -.204^*^ |
| (4) DASS-21 Anxiety | -.122^*^ | -.071 | -.196^*^ |
| (5) DASS-21 Stress | -.120^*^ | -.070 | -.132 |
| (6) DERS Non-acceptance | -.168^**^ | -.117 | -.200^*^ |
| (7) DERS Goals | -.191^***^ | -.122^*^ | -.287^**^ |
| (8) DERS Impulse | -.080 | -.002 | -.179 |
| (9) DERS Strategies | -.141^**^ | -.062 | -.231^*^ |
| (10) DERS Clarity | -.132^**^ | -.124^*^ | -.151 |
| (11) DERS Awareness | -.040 | .002 | -.132 |
| (12) ECR-R Anxiety | -.246^***^ | -.202^**^ | -.320^***^ |
| (13) ECR-R Avoidance | -.043 | -.053 | -.070 |

*TOS, Teruel Orthorexia Scale; HeOr, healthy orthorexia; OrNe, Orthorexia nervosa; DASS-21, Depression-Anxiety-Stress Scales; DERS, Difficulties in Emotion Regulation Scale; ECR-R, Experiences in Close Relationships – Revised. ***p<.001, **p<.01, *p<.05. r>0.2*

**Exclusion of participants > 60 years old**

**Supplementary Table 5**. Pearson correlations of variables under study for the subsample aged ≤ 60 years (*N* = 383).

| **Variable** | **correlation** | | | | | | | | | | | |
| --- | --- | --- | --- | --- | --- | --- | --- | --- | --- | --- | --- | --- |
|  | (1) | (2) | (3) | (4) | (5) | (6) | (7) | (8) | (9) | (10) | (11) | (12) |
| (1) TOS HeOr |  |  |  |  |  |  |  |  |  |  |  |  |
| (2) TOS OrNe | .464^***^ |  |  |  |  |  |  |  |  |  |  |  |
| (3) DASS-21 Depression | -.035 | .332^***^ |  |  |  |  |  |  |  |  |  |  |
| (4) DASS-21 Anxiety | .028 | .304^***^ | .670^***^ |  |  |  |  |  |  |  |  |  |
| (5) DASS-21 Stress | .024 | .341^***^ | .755^***^ | .671^***^ |  |  |  |  |  |  |  |  |
| (6) DERS Non-acceptance | .077 | .469^***^ | .489^***^ | .424^***^ | .545^***^ |  |  |  |  |  |  |  |
| (7) DERS Goals | -.017 | .286^***^ | .448^***^ | .409^***^ | .531^***^ | .529^***^ |  |  |  |  |  |  |
| (8) DERS Impulse | .062 | .442^***^ | .475^***^ | .395^***^ | .607^***^ | .671^***^ | .618^***^ |  |  |  |  |  |
| (9) DERS Strategies | -.019 | .404^***^ | .680^***^ | .566^***^ | .649^***^ | .722^***^ | .687^***^ | .731^***^ |  |  |  |  |
| (10) DERS Clarity | -.131^*^ | .278^***^ | .433^***^ | .336^***^ | .398^***^ | .502^***^ | .332^***^ | .469^***^ | .545^***^ |  |  |  |
| (11) DERS Awareness | -.199^***^ | .136^***^ | .262^***^ | .172^***^ | .204^***^ | .303^***^ | .110^*^ | .300^***^ | .325^***^ | .634^***^ |  |  |
| (12) ECR-R Anxiety | -.115^*^ | .216^***^ | .389^***^ | .375^***^ | .385^***^ | .369^***^ | .376^***^ | .334^***^ | .479^***^ | .366^***^ | .198^***^ |  |
| (13) ECR-R Avoidance | -.092 | .187^***^ | .334^***^ | .250^***^ | .219^***^ | .284^***^ | .137^**^ | .265^***^ | .322^***^ | .409^***^ | .436^***^ | .430^***^ |

*TOS, Teruel Orthorexia Scale; HeOr, healthy orthorexia; OrNe, Orthorexia nervosa; DASS-21, Depression-Anxiety-Stress Scales; DERS, Difficulties in Emotion Regulation Scale; ECR-R, Experiences in Close Relationships – Revised. ***p<.001, **p<.01, *p<.05. r>0.2*

**Supplementary Table 6.** Pearson correlations of variables under study in the male subsample aged ≤ 60 years (*N* = 120).

| **Variable** | **correlation** | | | | | | | | | | | |
| --- | --- | --- | --- | --- | --- | --- | --- | --- | --- | --- | --- | --- |
|  | (1) | (2) | (3) | (4) | (5) | (6) | (7) | (8) | (9) | (10) | (11) | (12) |
| (1) TOS HeOr |  |  |  |  |  |  |  |  |  |  |  |  |
| (2) TOS OrNe | .464^***^ |  |  |  |  |  |  |  |  |  |  |  |
| (3) DASS-21 Depression | -.277^**^ | .095 |  |  |  |  |  |  |  |  |  |  |
| (4) DASS-21 Anxiety | -.096 | .171 | .659^***^ |  |  |  |  |  |  |  |  |  |
| (5) DASS-21 Stress | -.196^*^ | .110 | .732^***^ | .738^***^ |  |  |  |  |  |  |  |  |
| (6) DERS Non-acceptance | .111 | .487^***^ | .420^***^ | .314^***^ | .454^***^ |  |  |  |  |  |  |  |
| (7) DERS Goals | -.037 | .370^***^ | .439^***^ | .399^***^ | .492^***^ | .579^***^ |  |  |  |  |  |  |
| (8) DERS Impulse | -.032 | .499^***^ | .344^***^ | .316^***^ | .488^***^ | .690^***^ | .584^***^ |  |  |  |  |  |
| (9) DERS Strategies | -.103 | .381^***^ | .645^***^ | .485^***^ | .589^***^ | .756^***^ | .711^***^ | .723^***^ |  |  |  |  |
| (10) DERS Clarity | -.224^*^ | .201^*^ | .417^***^ | .284^**^ | .404^***^ | .479^***^ | .329^***^ | .474^***^ | .533^***^ |  |  |  |
| (11) DERS Awareness | -.286^**^ | -.023 | .304^**^ | .159 | .217^*^ | .124 | -.019 | .198^*^ | .195^*^ | .651^***^ |  |  |
| (12) ECR-R Anxiety | -.277^**^ | .113 | .402^***^ | .388^***^ | .387^***^ | .373^***^ | .490^***^ | .331^***^ | .487^***^ | .292^**^ | .061 |  |
| (13) ECR-R Avoidance | -.209^*^ | .083 | .346^***^ | .166 | .200^*^ | .194^*^ | .099 | .267^**^ | .214^*^ | .325^***^ | .446^***^ | .234^*^ |

*TOS, Teruel Orthorexia Scale; HeOr, healthy orthorexia; OrNe, Orthorexia nervosa; DASS-21, Depression-Anxiety-Stress Scales; DERS, Difficulties in Emotion Regulation Scale; ECR-R, Experiences in Close Relationships – Revised. ***p<.001, **p<.01, *p<.05. r>0.2*

**Supplementary Table 7.** Pearson correlations of variables under study in the female subsample aged ≤ 60 years (*N* = 263).

| **Variable** | **correlation** | | | | | | | | | | | |
| --- | --- | --- | --- | --- | --- | --- | --- | --- | --- | --- | --- | --- |
|  | (1) | (2) | (3) | (4) | (5) | (6) | (7) | (8) | (9) | (10) | (11) | (12) |
| (1) TOS HeOr |  |  |  |  |  |  |  |  |  |  |  |  |
| (2) TOS OrNe | .467^***^ |  |  |  |  |  |  |  |  |  |  |  |
| (3) DASS-21 Depression | .067 | .411^***^ |  |  |  |  |  |  |  |  |  |  |
| (4) DASS-21 Anxiety | .066 | .340^***^ | .673^***^ |  |  |  |  |  |  |  |  |  |
| (5) DASS-21 Stress | .111 | .421^***^ | .765^***^ | .648^***^ |  |  |  |  |  |  |  |  |
| (6) DERS Non-acceptance | .054 | .465^***^ | .508^***^ | .442^***^ | .558^***^ |  |  |  |  |  |  |  |
| (7) DERS Goals | -.012 | .252^***^ | .448^***^ | .412^***^ | .543^***^ | .507^***^ |  |  |  |  |  |  |
| (8) DERS Impulse | .094 | .424^***^ | .515^***^ | .406^***^ | .637^***^ | .655^***^ | .630^***^ |  |  |  |  |  |
| (9) DERS Strategies | .009 | .412^***^ | .691^***^ | .584^***^ | .660^***^ | .703^***^ | .676^***^ | .727^***^ |  |  |  |  |
| (10) DERS Clarity | -.084 | .310^***^ | .441^***^ | .361^***^ | .404^***^ | .523^***^ | .334^***^ | .474^***^ | .559^***^ |  |  |  |
| (11) DERS Awareness | -.149^*^ | .206^**^ | .251^***^ | .189^**^ | .214^***^ | .398^***^ | .181^**^ | .357^***^ | .399^***^ | .627^***^ |  |  |
| (12) ECR-R Anxiety | -.036 | .256^***^ | .382^***^ | .373^***^ | .383^***^ | .368^***^ | .319^***^ | .333^***^ | .475^***^ | .401^***^ | .271^***^ |  |
| (13) ECR-R Avoidance | -.028 | .234^***^ | .340^***^ | .297^***^ | .254^***^ | .346^***^ | .166^**^ | .284^***^ | .392^***^ | .452^***^ | .429^***^ | .530^***^ |

*TOS, Teruel Orthorexia Scale; HeOr, healthy orthorexia; OrNe, Orthorexia nervosa; DASS-21, Depression-Anxiety-Stress Scales; DERS, Difficulties in Emotion Regulation Scale; ECR-R, Experiences in Close Relationships – Revised. ***p<.001, **p<.01, *p<.05. r>0.2*

**Partial correlations for the total sample**

**Supplementary Table 8**. Partial correlations, controlling for age, between study variables for the total sample.

| **Variable** | **partial correlation (Pearson)** | | | | | | | | | | | |
| --- | --- | --- | --- | --- | --- | --- | --- | --- | --- | --- | --- | --- |
|  | (1) | (2) | (3) | (4) | (5) | (6) | (7) | (8) | (9) | (10) | (11) | (12) |
| (1) TOS HeOr |  |  |  |  |  |  |  |  |  |  |  |  |
| (2) TOS OrNe | .465^***^ |  |  |  |  |  |  |  |  |  |  |  |
| (3) DASS-21 Depression | -.036 | .324^***^ |  |  |  |  |  |  |  |  |  |  |
| (4) DASS-21 Anxiety | .027 | .294^***^ | .666^***^ |  |  |  |  |  |  |  |  |  |
| (5) DASS-21 Stress | .023 | .332^***^ | .752^***^ | .666^***^ |  |  |  |  |  |  |  |  |
| (6) DERS Non-acceptance | .076 | .460^***^ | .481^***^ | .413^***^ | .536^***^ |  |  |  |  |  |  |  |
| (7) DERS Goals | -.019 | .271^***^ | .439^***^ | .396^***^ | .521^***^ | .513^***^ |  |  |  |  |  |  |
| (8) DERS Impulse | .061 | .438^***^ | .471^***^ | .389^***^ | .604^***^ | .669^***^ | .616^***^ |  |  |  |  |  |
| (9) DERS Strategies | -.020 | .395^***^ | .676^***^ | .558^***^ | .643^***^ | .716^***^ | .680^***^ | .729^***^ |  |  |  |  |
| (10) DERS Clarity | -.133^**^ | .267^***^ | .425^***^ | .325^***^ | .388^***^ | .491^***^ | .315^***^ | .464^***^ | .537^***^ |  |  |  |
| (11) DERS Awareness | -.200^***^ | .132^*^ | .260^***^ | .169^***^ | .201^***^ | .301^***^ | .104^*^ | .298^***^ | .323^***^ | .635^***^ |  |  |
| (12) ECR-R Anxiety | -.121^**^ | .196^***^ | .377^***^ | .358^***^ | .369^***^ | .343^***^ | .346^***^ | .325^***^ | .463^***^ | .347^***^ | .194^***^ |  |
| (13) ECR-R Avoidance | -.093 | .184^***^ | .331^***^ | .247^***^ | .216^***^ | .281^***^ | .132^***^ | .262^***^ | .320^***^ | .407^***^ | .435^***^ | .433^***^ |

*TOS, Teruel Orthorexia Scale; HeOr, healthy orthorexia; OrNe, Orthorexia nervosa; DASS-21, Depression-Anxiety-Stress Scales; DERS, Difficulties in Emotion Regulation Scale; ECR-R, Experiences in Close Relationships – Revised. ***p<.001, **p<.01, *p<.05. r>0.2*

**Supplementary Table 9.** Partial correlations, controlling for age, between study variables for the male sample.

| **Variable** | **partial correlation (Pearson)** | | | | | | | | | | | |
| --- | --- | --- | --- | --- | --- | --- | --- | --- | --- | --- | --- | --- |
|  | (1) | (2) | (3) | (4) | (5) | (6) | (7) | (8) | (9) | (10) | (11) | (12) |
| (1) TOS HeOr |  |  |  |  |  |  |  |  |  |  |  |  |
| (2) TOS OrNe | .477^***^ |  |  |  |  |  |  |  |  |  |  |  |
| (3) DASS-21 Depression | -.267^**^ | .074 |  |  |  |  |  |  |  |  |  |  |
| (4) DASS-21 Anxiety | -.083 | .152 | .645^***^ |  |  |  |  |  |  |  |  |  |
| (5) DASS-21 Stress | -.188^*^ | .096 | .726^***^ | .732^***^ |  |  |  |  |  |  |  |  |
| (6) DERS Non-acceptance | .130 | .477^***^ | .395^***^ | .286^**^ | .441^***^ |  |  |  |  |  |  |  |
| (7) DERS Goals | -.015 | .355^***^ | .406^***^ | .365^***^ | .479^***^ | .556^***^ |  |  |  |  |  |  |
| (8) DERS Impulse | -.018 | .490^***^ | .319^***^ | .291^**^ | .476^***^ | .679^***^ | .565^***^ |  |  |  |  |  |
| (9) DERS Strategies | -.088 | .367^***^ | .628^***^ | .461^***^ | .579^***^ | .744^***^ | .692^***^ | .712^***^ |  |  |  |  |
| (10) DERS Clarity | -.216^*^ | .187^*^ | .399^***^ | .263^**^ | .392^***^ | .464^***^ | .301^**^ | .460^***^ | .518^***^ |  |  |  |
| (11) DERS Awareness | -.279^**^ | -.039 | .286^**^ | .137 | .203^*^ | .100 | -.060 | .179 | .171 | .644^***^ |  |  |
| (12) ECR-R Anxiety | -.266^**^ | .081 | .363^***^ | .351^***^ | .367^***^ | .333^***^ | .438^***^ | .293^**^ | .448^***^ | .260^**^ | .020 |  |
| (13) ECR-R Avoidance | -.205^*^ | .075 | .340^***^ | .156 | .193^*^ | .184^*^ | .082 | .259^**^ | .204^*^ | .319^***^ | .442^***^ | .224^*^ |

*TOS, Teruel Orthorexia Scale; HeOr, healthy orthorexia; OrNe, Orthorexia nervosa; DASS-21, Depression-Anxiety-Stress Scales; DERS, Difficulties in Emotion Regulation Scale; ECR-R, Experiences in Close Relationships – Revised. ***p<.001, **p<.01, *p<.05. r>0.2*

**Supplementary Table 10.** Partial correlations, controlling for age, between study variables for the female sample.

| **Variable** | **partial correlation (Pearson)** | | | | | | | | | | | |
| --- | --- | --- | --- | --- | --- | --- | --- | --- | --- | --- | --- | --- |
|  | (1) | (2) | (3) | (4) | (5) | (6) | (7) | (8) | (9) | (10) | (11) | (12) |
| (1) TOS HeOr |  |  |  |  |  |  |  |  |  |  |  |  |
| (2) TOS OrNe | .465^***^ |  |  |  |  |  |  |  |  |  |  |  |
| (3) DASS-21 Depression | .065 | .410^***^ |  |  |  |  |  |  |  |  |  |  |
| (4) DASS-21 Anxiety | .063 | .335^***^ | .673^***^ |  |  |  |  |  |  |  |  |  |
| (5) DASS-21 Stress | .108 | .417^***^ | .765^***^ | .646^***^ |  |  |  |  |  |  |  |  |
| (6) DERS Non-acceptance | .048 | .458^***^ | .508^***^ | .438^***^ | .554^***^ |  |  |  |  |  |  |  |
| (7) DERS Goals | -.018 | .242^***^ | .448^***^ | .407^***^ | .540^***^ | .500^***^ |  |  |  |  |  |  |
| (8) DERS Impulse | .094 | .426^***^ | .516^***^ | .407^***^ | .638^***^ | .659^***^ | .635^***^ |  |  |  |  |  |
| (9) DERS Strategies | .006 | .409^***^ | .691^***^ | .582^***^ | .658^***^ | .702^***^ | .675^***^ | .729^***^ |  |  |  |  |
| (10) DERS Clarity | -.091 | .301^***^ | .440^***^ | .356^***^ | .399^***^ | .516^***^ | .323^***^ | .477^***^ | .556^***^ |  |  |  |
| (11) DERS Awareness | -.149^*^ | .207^**^ | .251^***^ | .190^**^ | .215^***^ | .401^***^ | .183^**^ | .357^***^ | .400^***^ | .632^***^ |  |  |
| (12) ECR-R Anxiety | -.047 | .241^***^ | .382^***^ | .367^***^ | .377^***^ | .354^***^ | .303^***^ | .340^***^ | .473^***^ | .387^***^ | .277^***^ |  |
| (13) ECR-R Avoidance | -.031 | .230^***^ | .339^***^ | .295^***^ | .251^***^ | .343^***^ | .161^**^ | .285^***^ | .390^***^ | .450^***^ | .430^***^ | .531^***^ |

*TOS, Teruel Orthorexia Scale; HeOr, healthy orthorexia; OrNe, Orthorexia nervosa; DASS-21, Depression-Anxiety-Stress Scales; DERS, Difficulties in Emotion Regulation Scale; ECR-R, Experiences in Close Relationships – Revised. ***p<.001, **p<.01, *p<.05. r>0.2*
